# Multiscale Modeling of Thermo–Electro–Mechanical Coupling of BGA Solder Joints in Microelectronic Systems of Ruggedized Computers for Signal Integrity Analysis

**DOI:** 10.3390/mi16111292

**Published:** 2025-11-18

**Authors:** Pan Li, Jin Huang, Jie Zhang, Hongxiao Gong, Jianjun Wang, Daijiang Zuo, Mengyang Su, Jiwei Shi

**Affiliations:** 1 State Key Laboratory of Electromechanical Integrated Manufacturing of High-Performance Electronic Equipments, Xidian University, Xi’an 710071, China; 23041212614@stu.xidian.edu.cn (P.L.); hxgong@xidian.edu.cn (H.G.); wangjianjun@xidian.edu.cn (J.W.); 23041212718@stu.xidian.edu.cn (D.Z.); 23041212582@stu.xidian.edu.cn (M.S.); 2 Zhongxing Telecommunication Equipment Corporation, Xi’an 710076, China; jiweishi2021@163.com

**Keywords:** BGA solder joints, multiscale, thermal–electrical–mechanical coupling modeling, signal integrity analysis

## Abstract

Ruggedized computers are the core of modern communication, guidance, control, and data-processing systems, and typically operate under extreme environmental conditions. However, under extreme service conditions such as temperature cycling, vibration, and mechanical shock, thermo–electro–mechanical (TME) multi-physics coupling in ball grid array (BGA) solder joints is particularly significant, severely affecting system reliability and signal integrity. To comprehensively elucidate the effects of thermal, electrical, and mechanical fields on solder joints and signal transmission, this study proposes a multiscale multi-physics modeling and analysis framework for BGA solder joints in microelectronic systems of ruggedized computers, covering the computer system level, motherboard level, solder joint level, and solder interconnect level. A model correlation study under ten thermal cycling conditions demonstrated an accuracy of 88.89%, confirming the validity and applicability of the proposed model. Based on this validated framework and model, the temperature distribution, stress–strain response, and signal integrity characteristics were further analyzed under combined conditions of thermal cycling, random vibration, and mechanical shock. The results indicate that a rise in temperature in solder joints induces thermal stresses and deformations, while variations in electrical conductivity under thermal loading trigger electromigration and concentration evolution, which further couple with stress gradients to form TME multi-physics interactions. Under such coupling, critical solder balls exhibit stress concentration at the metallurgical interfaces, with a maximum von Mises stress of 191.51 MPa accompanied by plastic strain accumulation. In addition, the PCIe high-speed interconnect experienced a maximum deformation of 16.104 μm and a voltage amplitude reduction of approximately 18.51% after 928 thermal cycles, exceeding the normal operating range. This research provides a theoretical basis and engineering reference for reliability assessment and optimization design of microelectronic systems in ruggedized computers in complex service environments.

## 1. Introduction

In recent years, with advances in chip and electronic component design and manufacturing technologies, electronic devices have been evolving toward higher density, greater integration, and miniaturization [1,2,3]. However, this trend has increasingly exposed electronic systems to coupled thermo–mechanical–electrical (TME) multi-physics problems under harsh environments, which have become key factors affecting system reliability [4,5,6,7,8]. Under harsh operating conditions, the combined effects of high temperature, mechanical vibration, and shock often lead to solder joint crack initiation and propagation, interconnection failure, and degradation of high-speed signal links, thereby substantially reducing the system’s overall reliability [9,10,11]. Kanai et al. [12] experimentally demonstrated that rapid thermal cycling induces equibiaxial stress in solder joints, leading to sequential formation and propagation of cross-shaped cracks; as crack density increases, mutual interference between cracks results in the formation of a fatigue crack network. Cheng et al. [13], using finite element analysis coupled with the Anand constitutive model, revealed the failure mechanisms of solder layers in high-power laser diode microchannel cooling systems under thermal cycling and random vibration, showing that thermal cycling predominantly drives evolution of damage and substantially limits reliability. Jeyun Yeom et al. [14] investigated the mechanical and microstructural degradation of solder joints made with different lead-free and SnPb solders under 3000 thermal cycles (−55 °C to +100 °C), revealing the correlation between crack and void evolution and shear strength degradation, thereby providing insights into developing highly reliable surface-mounted components for aerospace applications. Cui et al. [15] studied the electromigration (EM) reliability and failure mechanisms of complex electronic components, finding through simulations and experiments that interconnect reliability under EM is significantly influenced by the formation of solder voids, IMC growth, and crack evolution. Fan et al. [16] reported that the incorporation of Cu@Sn@Ag (CSA) core–shell particles into SAC305 solder joints significantly refines the microstructure and enhances tensile strength, while mitigating corrosion-induced degradation of mechanical properties over time. However, these studies primarily focused on single packages or isolated interconnects, often neglecting the system-level influence of coupled TME fields across the entire electronic assembly. As a result, the underlying mechanisms governing multiphysics-driven failure in complex systems, such as ruggedized computers, remain insufficiently understood.

In the field of multi-scale modeling, some researchers have proposed hierarchical modeling strategies for multi-chip packages and electronic components, in which models are established at the material, device, and system levels, and coupled computations are achieved through heterogeneous model transformation and information transfer [17,18]. Li et al. [19] investigated the reliability of PCB under thermal cycling and random vibration. Wei Yu et al. [20] presented how machine learning potentials and MD-to-FEM conversion facilitate multi-scale simulation with DFT accuracy. Wei Zheng et al. [21] proposed a reliability prediction method based on multi-physics coupling and hybrid-precision simulation, which integrates electro–thermal–mechanical analysis with IBIS-based topology modeling to reveal the failure mechanisms and signal integrity degradation of key CMOS chips in mixed-signal electronic systems under complex operating conditions, thereby significantly improving the accuracy of failure mode prediction and system reliability evaluation. Kaixiang Peng et al. [22] investigated the temperature-dependent electrical behavior of coaxial annular TSVs using a multi-physics approach, combining circuit and electromagnetic simulations, and proposed a metal–dielectric–metal TSV design to improve signal integrity across a wide temperature range. Although these studies improved modeling efficiency and cross-scale information transfer, they were mainly validated on simplified geometries and are difficult to extend to complex system-level assemblies containing chips, solder joints, and multi-layered motherboards.

In the field of multiphysics coupling analysis, Yang et al. [23] conducted thermo–mechanical coupling analysis of IGBT materials to predict the temperature distribution, equivalent stress, and equivalent strain of solder materials under actual operating conditions, thereby revealing the interaction mechanisms between thermal and mechanical effects. Chen et al. [24] experimentally and numerically characterized the thermoelectric coupling behavior of ternary lithium-ion batteries, demonstrating its critical influence on battery performance and lifetime. Xiao et al. [25] systematically studied the fatigue failure mechanisms and lifetime prediction methods of electronic devices under random vibration environments, proposing an integrated evaluation approach combining finite element analysis with experimental validation. Gao et al. [26] explored the modeling and signal integrity analysis of silicon interposer channels, providing insights into the challenges and solutions in high-speed interconnects. In summary, existing research has mainly focused on single-field coupling, such as thermo–mechanical, thermo–electrical, and electro–mechanical interactions [27,28,29,30,31], whereas comprehensive investigations of fully coupled thermo–electro–mechanical effects within high-density electronic systems are scarce. Furthermore, for ruggedized computers, direct full-scale modeling of all hierarchical structures would dramatically increase computational cost and model complexity [32].

Therefore, it is essential to develop an efficient, scalable, and accurate multiscale TME modeling and analysis framework to provide deeper insight into Multiphysics interactions and to support both signal integrity (SI) and reliability assessments under extreme operating conditions.

The main contributions of this study are summarized as follows:

(1) It focuses on solder joints and interconnect structures under thermo–electro–mechanical (TME) Multiphysics coupling;
(2) It proposes a multi-scale modeling strategy spanning solder joint, board, and system-level analysis;
(3) It investigates microstructural evolution in solder joints and interconnects and its impact on signal integrity;
(4) It provides experimental validation and optimization insights, offering guidance for enhancing the reliability and performance of ruggedized computers.

## 2. Modeling

To accurately characterize the Multiphysics coupling behavior of electronic packaging structures under complex operating conditions, a Multiphysics coupling mathematical model is established in this section, together with a numerical information transfer method between heterogeneous models. The constructed coupling model encompasses typical physical interactions, including thermal–electrical, thermal–mechanical, and mechanical–electrical couplings, while incorporating atomic concentration migration effects to provide a theoretical basis for elucidating the interactions among thermal, electrical, and mechanical fields. Based on this framework, a heterogeneous model coupling strategy is further proposed, which includes substructure-based multiscale modeling, deformation information transfer, and multiscale simplification techniques. These methods enable efficient exchange of information between different physical fields and across multiple scales, balancing modeling accuracy with computational efficiency.

### 2.1. Multiphysics Coupling Mathematical Model

**Figure 1:**
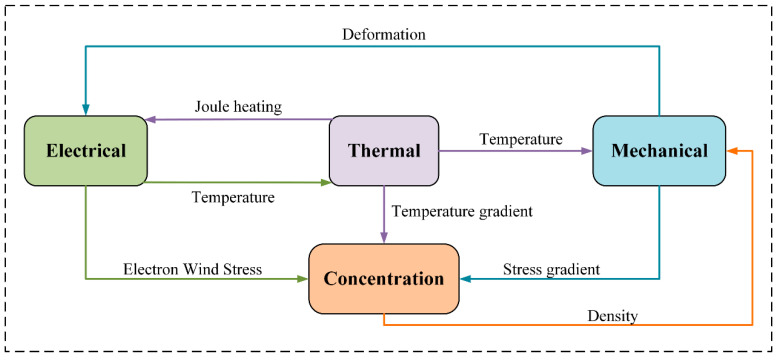
Coupling relationships among multiple physical fields.

During the operational process of ruggedized computers, the coupled effects of thermal, mechanical, and electrical fields are highly pronounced, significantly affecting system reliability. In operation, the interactions among local heating, current distribution, and stress variations give rise to complex multiscale coupling phenomena. Therefore, conducting a systematic theoretical analysis of the Multiphysics coupling behavior of BGA solder balls is of great significance. This study mainly focuses on the interactions among the electric field, temperature field, stress field, and charge concentration field. As shown in Figure 1, complex interactions exist among the electrical, thermal, mechanical, and concentration fields. The electrical field induces a local temperature rise through Joule heating and generates electron wind stress in the conductor. The thermal field is not only influenced by the electrical field but also affects the mechanical and concentration fields through temperature and temperature gradients. The mechanical field, driven by temperature, produces stress and deformation, and further develops stress gradients that drive the migration of the concentration field. The evolution of the concentration field is jointly influenced by both stress and temperature gradients, while its density variation, in turn, feeds back to the mechanical field. Such Multiphysics coupling relationships collectively determine the evolution behavior and service reliability of solder joints.

#### 2.1.1. Thermo–Electrical Coupling Effect

During the operation of the computer, key components such as the CPU, DDR, and GPU generate significant Joule heat during data processing, resulting in local temperature rises. Meanwhile, the electrical conductivity of conductive materials on the PCB, including copper traces and solder joints, is temperature-dependent. Variations in temperature lead to changes in conductivity, which in turn affect the distribution of current density and electric field. The fundamental coupling between the thermal field (temperature field) and the electrical field is governed by the conservation of energy—including Joule heating and thermoelectric effects such as Peltier and Seebeck—and the conservation of charge, which accounts for temperature-dependent conductivity:

Charge conservation equation:

(1) $$\nabla \cdot \left[\sigma (T)\left(-\nabla \phi +S(T)\nabla T\right)\right]=0$$

where *Φ* is the electric potential, *σ*(*T*) is the temperature-dependent electrical conductivity, *S*(*T*) is the Seebeck coefficient, and *T* denotes the temperature.

Energy Conservation Equation:

(2) $$\rho c_{p}\frac{\partial T}{\partial t}=\nabla \cdot \left(k(T)\nabla T\right)+\frac{J\cdot J}{\sigma (T)}+\nabla \cdot \left(S(T)TJ\right)+Q_{\mathrm{mech}}$$

where *ρ* is the material density, *c_p_* is the specific heat capacity, *k*(*T*) is the temperature-dependent thermal conductivity, *Q*_mech_ is the power density generated by mechanical dissipation converted to heat, and J denotes the current density.

The effect of the temperature field on the electric field is expressed by Equation (3):

(3) $$R=\left[\rho_{0}(1+\alpha (T-T_{0}))\right]$$

where *R* is the electrical resistance, *T* is the temperature, and *ρ*_0_ is the conductor resistance at the reference temperature. Physically, this indicates that as the temperature of BGA solder joints increases, their electrical resistance correspondingly rises.

Furthermore, during high-speed signal transmission, the skin effect and dielectric losses cause heating of the computer traces, further exacerbating the nonuniformity of the temperature field. Localized overheating can affect the clock stability of the chips and may even lead to performance degradation or permanent damage to electronic components. The influence of the electric field on the temperature field is expressed by Equation (4):

(4) $$Q=\sigma_{c}{\left|\nabla V\right|}^{2}$$

where *Q* is the Joule heat, *σ_c_*is the electrical conductivity, and *V* denotes the electric potential. Physically, this means that when a conductor on the computer carries current, the generated Joule heat is proportional to the square of the electric potential gradient and also proportional to the electrical conductivity of the conductor.

#### 2.1.2. Thermo–Mechanical Coupling Effect

During temperature variations in the computer, various components undergo thermal expansion or contraction [20]. Due to the mechanical constraints inherent in computer structures, thermal deformation of the board is restricted, generating internal thermal stress. Furthermore, complex PCB comprises multiple materials such as copper, solder, and FR-4, which have different coefficients of thermal expansion (CTE), leading to stress gradients among components during thermal cycling. For instance, between the chip and PCB, the mismatch of CTEs between the solder and PCB can induce fatigue-induced cracks in solder joints under repeated temperature variations, reducing soldering reliability. Such thermal–mechanical coupling effects may also deform high-speed signal paths, thereby affecting signal integrity.

The effect of the temperature field on the structural field is expressed by Equation (5).

(5) $$\sigma_{ij}=E_{ijkl}\varepsilon_{kl}-\beta_{kl}(T-T_{0})\varepsilon_{kl}$$

where *σ* is the stress, *ε* is the strain, *E* is the elastic modulus, *β* is the thermal expansion coefficient, and T denotes the temperature. Physically, temperature differences within the computer structure cause deformations of the elements, which in turn generate stress. In multi-physics coupling analysis, the feedback effect of the structural field on the thermal field is generally weak. In engineering practice, a one-way coupling assumption is often adopted to improve computational efficiency.

#### 2.1.3. Mechanical–Electrical Coupling Effect

In practical operating environments, computer may be subjected to external vibrations or shocks. Due to the differing elastic moduli of components within the board-level circuitry, nonuniform mechanical loading can induce localized stress concentrations, leading to changes in electrical resistance. The influence of the structural field on the electrical resistance in the electric field can be expressed by Equation (6).

(6) $$R(\varepsilon ,T)\approx R_{0}\left(1+\kappa_{\rho }\Delta T+G_{\varepsilon }\varepsilon \right)$$

where *R*_0_ is the initial resistance; *κ*_*ρ*_ is the temperature coefficient of resistance; Δ*T* is the temperature change; *G*_*ε*_ is the gauge factor relating resistance to strain; and *ε* denotes the linear strain. Physically, variations in strain within the structural field induce corresponding changes in electrical resistance.

When the computer is subjected to mechanical impact, solder joints and interconnects may undergo slight deformations. Under the combined influence of electric field, temperature, stress, and concentration gradients, metal atoms can migrate, alter current paths and locally increase current density, which may lead to electromigration. Driven by multiple migration forces, metal atoms within the solder joint move directionally, resulting in the formation of voids. Such changes impede current flow, restrict electrical transmission at the board level, and can ultimately cause circuit failure [33].

Furthermore, high current density may induce migration of metal atoms within the solder joints, resulting in void formation that compromises the mechanical strength of the joints. After prolonged service, solder joints may crack or fail due to electromigration. The influence of the electric field on the structural field can be expressed by Equation (7):

(7) f=ρE+J×B

where **f** is the force vector per unit volume acting on the charge, ρ is the free charge density, **E** is the electric field strength, **J** is the current density vector and **B** is the magnetic flux density vector. Physically, the combined effects of the electric and magnetic fields exert forces on the conductors within the computer, thereby influencing their mechanical behavior.

#### 2.1.4. Concentration-Driven Atomic Migration

Atoms undergo directional motion under the combined effects of electron wind, thermal gradients, and stress gradients. As electromigration progresses, it leads to an imbalance in atomic concentration distribution. According to Le Chatelier’s principle, such nonuniform concentration generates a driving force that encourages atoms to move toward regions of lower concentration, thereby mitigating electromigration. The influence of this effect increases with the magnitude of the concentration gradient. Migration driven by atomic concentration gradients is referred to as chemical migration. For the concentration of metal atoms, the atomic diffusion flux can be expressed as follows:

(8) $$\frac{\partial C}{\partial t}+\nabla \cdot J_{c}=R_{V}$$

(9) $$J_{c}=-D\nabla c+\frac{DZ^{*}e\rho }{k_{B}T}CJ-\frac{D\Omega C}{k_{B}T}\nabla \sigma_{H}-DC\frac{Q^{*}}{k_{B}T^{2}}\nabla T$$

where ∇c is the concentration gradient; D is the atomic diffusion coefficient; $Z^{*}$ is the effective charge number; e is the elementary charge; Ω is the atomic volume; $\sigma_{H}$ is the hydrostatic stress; $Q^{*}$ is the activation energy for thermal migration; $k_{B}$ is the Stefan-Boltzmann constant; and *T* is the absolute temperature. The migration force acts in the direction of decreasing concentration.

Therefore, electromigration failure results from the combined action of driving forces such as electron wind, thermal gradients, stress gradients, and atomic concentration gradients. Consequently, the study of electromigration mechanisms should consider all these driving factors comprehensively, necessitating a multi-physics coupling analysis.

### 2.2. Numerical Information Transfer Method for Heterogeneous Model Coupling

In multi-physics coupling analysis, multiple data sources exhibit diverse types and complex interaction pathways, and uncertainties arising from these sources can significantly affect performance evaluation. On one hand, system-level performance assessment typically involves multidisciplinary analyses, such as thermal, mechanical, and electrical studies at different scales. The substantial differences in model formulations across disciplines and the heterogeneity of numerical solution schemes make the matching and sharing of mesh information between heterogeneous models challenging. Interaction of nodal information across physical fields must be based on unified numerical transfer rules, with mesh alignment serving as a prerequisite for ensuring data consistency. On the other hand, the types of coupled information are diverse, and not all data can be directly transferred via nodal information. Integrating and fusing certain information is complex and requires collaborative numerical methods to achieve indirect transfer.

#### 2.2.1. Substructure Theory for Multiscale Modeling

In the field of finite element multiscale modeling, substructuring techniques are widely employed as a core method for packaging structure analysis. In this study, based on the structural characteristics of packaging components, the chip units and solder assemblies are identified as the core regions for substructure generation. The technical workflow mainly includes the following steps: First, a three-dimensional geometric model of the chip–solder composite structure is constructed using parametric modeling techniques. Next, a multiscale mesh generation strategy is established, ensuring mesh compatibility across scales through size control. On this basis, the determination of material constitutive parameters is emphasized, and an equivalent elastic mechanical parameter derivation method based on microstructural features is proposed, establishing a modulus correction model under multi-physics coupling conditions. Additionally, corresponding programs are developed to implement the substructuring of chip solder joints, enabling automated generation.

The substructure method can be regarded as an extension of the element concept. When the internal degrees of freedom are not condensed, a single substructure is equivalent to a “super element” containing numerous internal degrees of freedom. Prior to integrating the substructure into the global computation, the internal degrees of freedom are condensed to reduce the overall system size, retaining only the external nodes required for connection with other elements. The substructure method introduces the concepts of master degrees of freedom (DOF) nodes and slave DOF nodes. Master DOF nodes are those that connect the substructure element to other elements, while slave DOF nodes are those whose DOFs need to be condensed. Based on this concept, with appropriate node numbering, the stiffness matrix of the substructure, along with the corresponding nodal displacement and load vectors, can be expressed in the form shown in Equation (10):

(10) $$\left[\begin{array}{cc} K_{nm} & K_{ms}\\ K_{sm} & K_{ss} \end{array}\right]\left[\begin{array}{c} a_{m}\\ a_{s} \end{array}\right]=\left[\begin{array}{c} F_{m}\\ F_{s} \end{array}\right]$$

where $a_{m}$ is the displacement vector of the master DOF nodes, and $a_{s}$ is the displacement vector of the slave DOF nodes.

Based on Equation (10), the resulting expressions are given in Equations (11) and (12):

(11) $$K_{mm}a_{m}+K_{ms}a_{s}=F_{m}$$

(12) $$K_{sm}a_{m}+K_{ss}a_{s}=F_{s}$$

By rearranging Equation (12), Equation (13) can be obtained.

(13) $$a_{s}={K_{ss}}^{-1}(F_{s}-K_{sm}a_{m})$$

By substituting Equation (13) into Equation (11), Equation (14) is obtained.

(14) $$K_{mm}a_{m}+K_{ms}[{K_{ss}}^{-1}(F_{s}-K_{sm}a_{m})]=F_{m}$$

By rearranging Equation (14), Equation (15) can be obtained.

(15) $$(K_{mm}-K_{ms}{K_{ss}}^{-1}K_{sm})a_{m}=F_{m}-K_{ms}{K_{ss}}^{-1}F_{s}$$

By defining $K^{*}=K_{mn}-K_{ms}{K_{ss}}^{-1}K_{sm}$, $F^{*}=F_{m}-K_{ms}{K_{ss}}^{-1}F_{s}$, the condensed equation can be expressed in the simplified form shown in Equation (16)*:*

(16) $$K^{*}a_{m}=F^{*}$$

This method reconstructs the equilibrium equations via a stiffness matrix reduction algorithm, reducing the computational burden of element matrices while maintaining the accuracy of the original system’s mechanical response. Based on this approach, a hierarchical substructure solution framework is established, which combines multilevel substructure decomposition with parallel computing architecture, enabling efficient handling of large-scale engineering models with millions of degrees of freedom and substantially improving computational efficiency.

#### 2.2.2. Unified Error-Bound Mapping Method

In the multi-physics analysis of BGA solder joints in Ruggedized Computers, efficient transfer of deformation information across structural, thermal, and electromagnetic domains is critical. However, the solver architectures of these domains are inherently heterogeneous—structural solvers adopt finite element methods (FEM) with hybrid solid–shell elements, electromagnetic solvers employ frequency-domain FEM, time-domain finite-difference, or method-of-moments schemes, and thermal solvers use hexahedron-dominated finite volume meshes. Such numerical heterogeneity results in grid incompatibility, making unified cross-field data transfer highly challenging.

To overcome this issue, a unified error-bound mapping method is proposed as the core of this work. This method quantitatively establishes the correlation between mesoscale equivalent layers in mechanical deformation fields and electromagnetic signal integrity (SI) responses, enabling traceable, verifiable data propagation across physical domains.

Instead of directly interpolating deformation data, the proposed mapping introduces an error-bound model that constrains deformation–field translation within a defined tolerance range. By tracking equivalent nodal displacements and local strain energy densities at each layer interface, the framework evaluates cumulative deviations during the deformation-to-SI transformation. This quantitative verification bridges mechanical deformation and electromagnetic signal behavior, providing a physically consistent correlation that replaces empirical adjustment with rigorous cross-domain validation.

The framework integrates Ansys SpaceClaim2023 R1, Workbench2023 R1, Icepak2023 R1, AEDT2023 R1, and CST Studio Suite2023 to support this mapping process. Parametric geometries are first constructed in SpaceClaim; structural responses are computed in Workbench; thermal conditions are evaluated in Icepak; and electromagnetic characteristics are analyzed in AEDT and CST. Standardized data interfaces automate the transfer of geometric and physical quantities among solvers.

A cross-reference table (Table 1) summarizes the detailed layer-wise mapping and verification workflows that support the proposed unified error-bound mapping method. The table outlines how deformation and field data are transferred and validated across geometric, thermal, thermo–mechanical, and electromagnetic layers. Specifically, geometry updating and solid–shell equivalence checks ensure structural consistency; temperature field mapping and thermal boundary verification maintain thermo–structural coherence; deformation-to-electromagnetic mapping with stress–strain verification secures mechanical–electrical correlation; and electromagnetic field–geometry consistency together with S-parameter validation (S11, S21) guarantees the accuracy of final signal integrity analysis.

This structured mapping and verification process ensures that deformation, thermal, and electromagnetic responses are physically correlated within an established error bound. The proposed unified error-bound mapping framework thus transforms multiphysics co-simulation from qualitative coupling into a quantitatively validated, reproducible methodology—offering a robust foundation for reliability assessment of Ruggedized Computers under complex service conditions.

#### 2.2.3. Simplification Method for Multiscale Analysis of Heterogeneous Models

**Figure 2:**
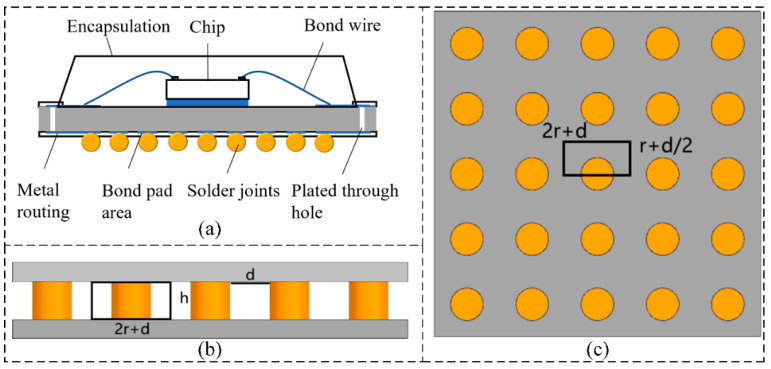
Cross-sectional model and solder ball equivalence of flip-chip BGA. (**a**) Cross-sectional model of flip-chip BGA; (**b**) Normal equivalence of solder balls. (**c**) Lateral equivalence of solder balls.

In the multiscale integrated analysis process, the key step is to identify critical local regions after the macroscopic-scale analysis and to perform equivalent simplification for these regions. In this study, an equivalence approach based on material properties is adopted, where a homogenized solid replaces the entire solder ball layer, possessing equivalent material parameters. Figure 2a illustrates a schematic cross-sectional model of a flip-chip BGA. This section provides a detailed description of the material property equivalence theory for BGA solder balls, including the simplification methods for equivalent density, equivalent specific heat capacity, and equivalent thermal conductivity.

Assume that the chip solder ball is a cylinder with a base radius of *r*, and that both the equivalent layer height and the solder ball height are ℎ. Subscripts *b* and *e* denote the solder ball and the equivalent layer, respectively, while subscript *a* refers to the filler or air surrounding the solder ball. *V* represents volume, and *A* represents base area. The volume ratio can be expressed as follows:

(17) $$\varphi =\frac{V_{b}}{V_{e}}=\frac{A_{b}}{A_{e}}$$

where φ is the volume fraction, $V_{b}$ is the volume of the solder ball layer, $V_{e}$ is the equivalent volume of the solder ball layer, $A_{b}$ is the base area of the solder ball layer, and $A_{e}$ is the equivalent base area of the solder ball layer. The equivalent volume is given by $V_{e}=V_{a}+V_{b}$.

(1) According to the principle of mass conservation, the density of the equivalent material is calculated using a volume-weighted averaging method and can be expressed as follows:
  (18) $$\rho_{e}=\frac{m_{e}}{V_{e}}=\frac{m_{a}+m_{b}}{V_{b}/\varphi }=(1-\varphi )\rho_{a}+\varphi \rho_{b}$$
  where $\rho_{e}$ is the equivalent density of the solder ball layer, $m_{e}$ is the equivalent mass of the solder ball layer, $m_{a}$ and $m_{b}$ are the masses of the surrounding filler and the solder ball, respectively, and $\rho_{a}$ is the density of the filler. If the filler material (e.g., air) is neglected, the equivalent density simplifies to $\rho_{e}=\varphi \rho_{b}$.
(2) The derivation of the equivalent specific heat capacity is also based on the volume-weighted averaging method and can be expressed as follows:
  (19) $$C_{e}=\frac{C_{a}m_{a}+C_{b}m_{b}}{m_{e}}=(1-\varphi )C_{a}\frac{\rho_{a}}{\rho_{e}}+\varphi C_{b}\frac{\rho_{B}}{\rho_{e}}$$
  where $C_{e}$ is the equivalent specific heat capacity of the solder ball layer, and $C_{a}$ and $C_{b}$ are the specific heat capacities of the surrounding filler and the solder ball, respectively. If the filler material (e.g., air) is neglected, the equivalent specific heat capacity simplifies to $C_{e}=C_{b}$.
(3) Since the solder balls exhibit anisotropic distribution in both the in-plane and normal directions, the equivalent thermal conductivity must be considered separately for the normal and lateral directions. In the normal direction, the thermal conduction path of the solder balls can be simplified as a series model. Figure 2b shows the normal equivalence schematic of the solder balls. Considering a subunit from the figure, with solder ball spacing *d*, and assuming the thermal conductivity of the surrounding filler (or air) is $\lambda_{a}$ and that of the solder ball is $\lambda_{a}$, the equivalent thermal conductivity in the normal direction can be expressed as follows:
  (20) $$\lambda_{ez}=\frac{\lambda_{a}A_{a}+\lambda_{b}A_{b}}{A_{e}}=(1-\varphi )\lambda_{a}+\varphi \lambda_{b}$$

If the filler material (e.g., air) is neglected, the equivalent thermal conductivity in the normal direction simplifies to $\lambda_{ez}=\varphi \lambda_{b}$.

In the lateral direction, the thermal conduction path of the solder balls can be simplified as a parallel model. The lateral equivalence of the solder balls is shown in Figure 2c. The equivalent thermal conductivity in the lateral direction can be expressed as follows:

(21) $$\lambda_{ex}=\frac{\sqrt{\varphi }\lambda_{a}\lambda_{b}+(1-\varphi ){\lambda_{a}}^{2}}{(\sqrt{\varphi }-\varphi )\lambda_{b}+(1-\sqrt{\varphi }+\varphi )\lambda_{a}}$$

If the filler material (e.g., air) is neglected, the equivalent thermal conductivity in the lateral direction simplifies to $\lambda_{ex}=0$

### 2.3. Multiscale Model of Ruggedized Computers

**Figure 3:**
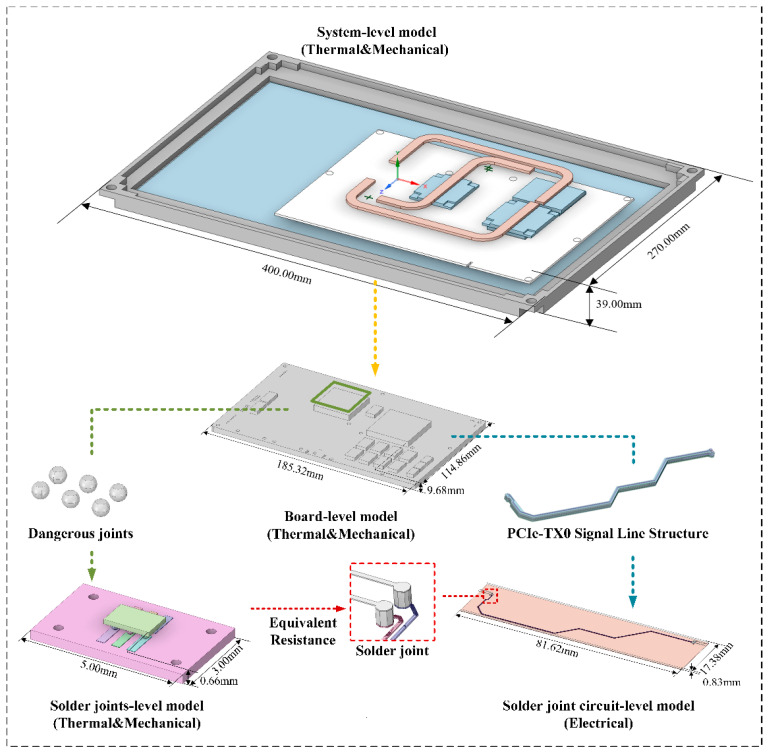
Multiscale multi-physics model of the Ruggedized Computer.

To verify the proposed efficient heterogeneous model coupling and multiscale modeling method, the ruggedized computer was selected as the research subject. Using parametric modeling techniques, a multiscale model of the ruggedized computer was constructed on the Ansys SpaceClaim platform, including the system-level model, the board-level model, the mesoscopic solder joint-level model, and the solder joint circuit-level model, as shown in Figure 3. The system-level model adopts a sealed design that isolates the fan from the internal chassis. The simplified chassis model mainly consists of the display, motherboard, CPU, GPU, bridge chip, copper heat pipes, and aluminum heat sinks. The motherboard model is based on the LS3A5000 board. The mesoscopic solder joint model selects six critical solder joints from regions of high thermal stress concentration on the motherboard, with BGA solder balls of 0.5 mm diameter, 1 mm spacing, and 0.3 mm height. The solder joint–trace model specifically selects a pair of differential PCIe lines (PCIe_TX0_P/N) from the PCB file and constructs the corresponding connected chip solder joint model. The material properties of the models are listed in Table 2 [34,35]. The temperature-dependent elastic modulus (*Y*), Poisson’s ratio (*ν*), and coefficient of thermal expansion (CTE) of Sn–3.0Ag–0.5Cu (wt.%) vary with temperature as shown in Table 3. Considering that the melting point of SAC305 solder is approximately 217 °C, the thermal cycling range in this study (−55 °C to 150 °C) exceeds half of the melting temperature. It is imperative to account for the creep characteristics of the solder. In this context, the Anand viscoplastic unified model is selected to characterize the viscoplastic behavior of the solder. Table 4 presents the nine pertinent parameters of the Anand viscoplastic unified model for Sn–3.0Ag–0.5Cu (wt.%).

The multi-level temperature cycling load is transmitted following the “chassis–motherboard–solder joint” hierarchical coupling principle, achieving multiscale correlation through stepwise mapping of thermal boundary conditions.

(1) System-Level Thermal Cycling Load

The complete chassis model transfers heat through natural convection and radiation. The natural convection heat transfer coefficient *h* (W·m^−2^·K^−1^) is determined using the empirical correlation [36] derived from the Rayleigh–Nusselt relation, as expressed in Equation (22). The detailed empirical correlations for natural convection are provided in Appendix A. The coefficient *C* = 1.31 W·m^−2^·K^−4/3^ is selected based on the correlation for natural convection over a vertical plate:

(22) $$h=C{\left(T_{s}-T_{amb}\right)}^{1/3}$$

where $T_{s}$ is the surface temperature and $T_{amb}$ is the ambient temperature.

Radiative heat transfer is calculated using the Stefan–Boltzmann law [37], as expressed in Equation (23):

(23) $$q_{rad}=\varepsilon \sigma (T_{s}^{4}-T_{amb}^{4})$$

In Equation (23), the emissivity is taken as 0.85, and the Stefan–Boltzmann constant is 5.67 × 10^−8^ W/(m^2^·K^4^). The chip heat sources are applied with non-uniform distribution: the CPU center region has a heat flux density of 15 W/cm^2^, while the DDR memory is uniformly loaded with 4.2 W.

(2) Board-Level Thermal Cycling Load

**Figure 4:**
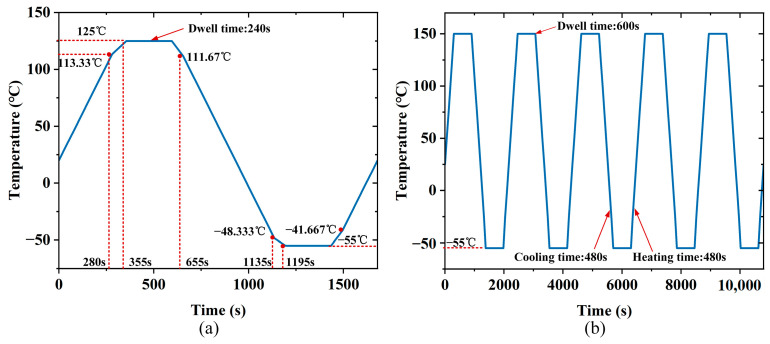
Boundary conditions: (**a**) Motherboard temperature cycling profile. (**b**) Solder joint temperature cycling profile.

The transient temperature field at the motherboard mounting surface is extracted from the chassis model and used as the input boundary condition, onto which the thermal cycling load is superimposed. The temperature loading function is expressed as follows:

(24) $$T_{load}(t)=T_{chassis}(t)+T_{stand}(t)$$

where *T_chassis_*(*t*) is the chassis temperature time history, and *T_stand_*(*t*) represents the standard cyclic component. The detailed thermal cycling profile is shown in Figure 4a. The simulation starts at an ambient temperature of 20 °C. Each cycle consists of a 1200 s transition phase to capture the transient temperature change, followed by a 240 s dwell at 125 °C to represent high-temperature operating conditions, and a 240 s dwell at −55 °C to simulate thermal contraction under extreme cold conditions.

(3) Solder Joint–Trace Level Thermal Load Mapping

Using the Ansys sub-modeling technique, the motherboard-level temperature field was interpolated and mapped onto the mesoscopic solder joint model. To ensure consistency in boundary temperature transfer, a cubic spline interpolation algorithm was employed. To quantitatively evaluate the mapping accuracy, temperature data were sampled at corresponding nodes along critical component boundaries (GPU, PCIe connector, and DDR regions) in both the system-level and board-level models. The results show that the root-mean-square (RMS) error was 2 °C, confirming that the interpolation error remained within 2 °C. The fitted thermal cycling curve is shown in Figure 4b.

(4) Mechanical Loads

Mechanical loads interact with the temperature field through sequential coupling. First, a transient thermomechanical analysis is performed, and the resulting temperature field is applied to the structural domain as thermal strain load. The resulting thermal deformation is used as the initial state, followed by the sequential application of random vibration and mechanical shock loads. A three-axis PSD vibration spectrum, as listed in Table 5, is applied at the motherboard mounting holes, and a trailing peak sawtooth mechanical shock load, as listed in Table 6, is applied on the six faces of the full system.

**Figure 5:**
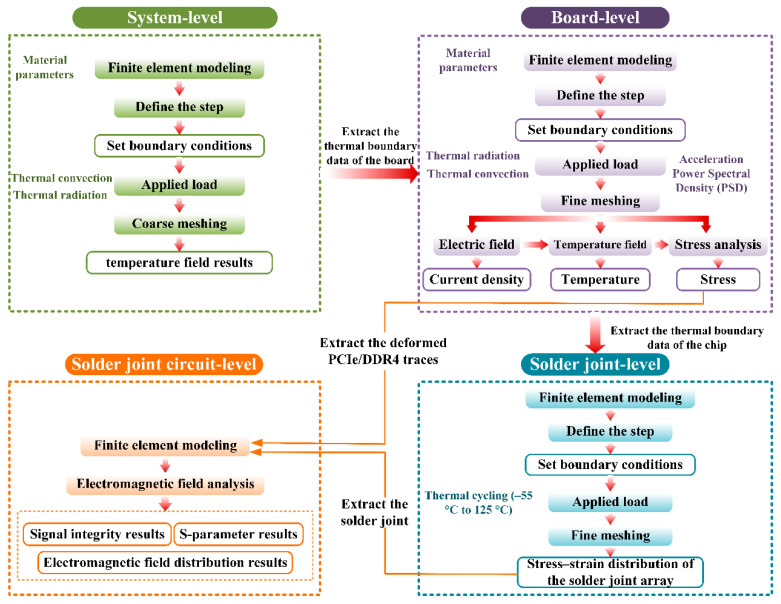
Flowchart of the multiscale analysis.

To overcome the limitations of analyzing a single solder ball, which cannot fully capture the system-level multi-physics coupling behavior, a multiscale integrated analysis method is proposed. First, the model is divided into four hierarchical levels according to the required analysis granularity. Second, methods for transferring data between mesh nodes at different scales are developed to ensure compatibility and consistency across the multiscale simulation process. Finally, based on performance evaluation requirements, a multi-level macro-to-mesoscopic simulation analysis is conducted to assess the thermo–mechanical–electrical coupled behavior of BGA solder joints under complex service environments, as illustrated in Figure 5.

#### Model Validation

**Figure 6:**
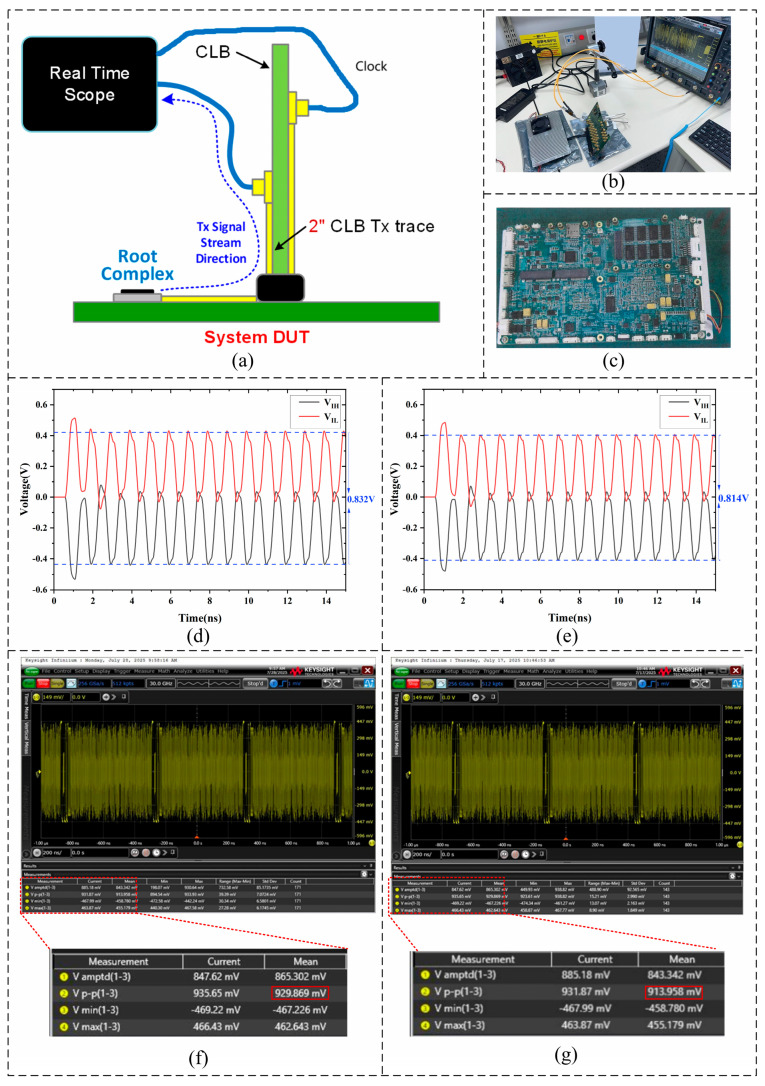
Experimental Test: (**a**) Schematic of the experimental setup. (**b**) Panoramic view of the test platform (Oscilloscope configuration: Keysight real-time oscilloscope, 30 GHz bandwidth, 256 GSa/s sampling rate, 50 Ω termination; trigger on Tx positive edge; 20 GHz low-pass digital filter applied for noise suppression). (**c**) The system motherboard used for testing. (**d**) PCIe_TX0_P/N port voltage distribution before 10 thermal cycles. (**e**) PCIe_TX0_P/N port voltage distribution after 10 thermal cycles. (**f**) Experimental results before 10 thermal cycles. (**g**) Experimental results after 10 thermal cycles.

To verify the model correlation at 10 thermal cycles, a test platform was established as shown in Figure 6a. The test system consists of a Root Complex, a Carrier Load Board (CLB), and a real-time oscilloscope. The PCIe CEM transmitter voltage test was performed using the compliance load board (CLB) connected to the system DUT through a 2-inch Tx trace, as illustrated in Figure 6a. Figure 6d,e compare the voltage waveforms at different ports before and after 10 thermal cycling: the peak-to-peak voltage of the differential signal at the receiver decreased from 0.832 V to 0.814 V, corresponding to a voltage drop (Δ*V_sim_*) of 0.018 V. Similarly, Figure 6f,g show the oscilloscope-captured time-domain signals, where the peak-to-peak voltage decreased from 0.929 V to 0.913V, a drop of 0.016 V(Δ*V_exp_*) after 10 cycles.

The simulated voltage attenuation was compared with the experimental measurement, and the model accuracy was calculated using the voltage drop magnitudes (Δ*V_sim_* − Δ*V_exp_*), representing the relative error of the peak-to-peak differential voltage between simulation and experiment under 10 thermal cycles, where the reference channel is the PCIe differential receiver signal measured on the compliance load board (CLB) through a 50 Ω terminated coaxial channel.

(25) $$\mathrm{Model}\;\mathrm{Accuracy}=\left(1-\frac{\left|\Delta V_{\mathrm{sim}}-\Delta V_{\exp}\right|}{\Delta V_{\exp}}\right)\times 100\%$$

where Δ*V_sim_*—voltage drop magnitude predicted by the numerical simulation, Δ*V_exp_*—voltage drop magnitude obtained from experimental measurement.

Using this definition, the model accuracy at 10 thermal cycles was calculated to be 88.89%. This result indicates that the multi-physics coupled model established in this work exhibits good accuracy.

## 3. Results and Discussion

### 3.1. Multilevel Simulation Analysis

**Figure 7:**
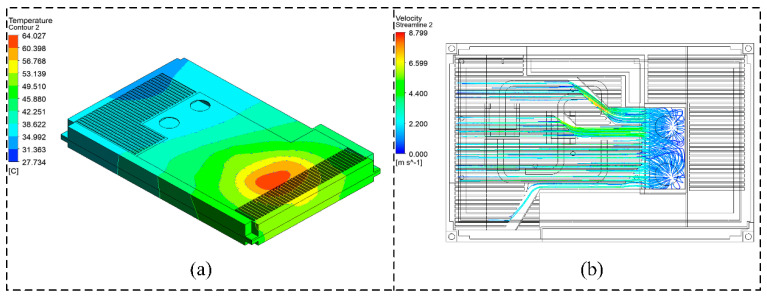
System-level temperature distribution results: (**a**) Temperature distribution cloud at ambient conditions. (**b**) Forced airflow field distribution.

The system-level thermal simulation results indicate that under ambient conditions, the chassis reaches thermal equilibrium through natural convection and forced air cooling. As shown in Figure 7a, the temperature distribution contour of the chassis under ambient conditions is presented, while Figure 7b illustrates the airflow distribution under forced cooling. The temperature field of the chassis exhibits a significant spatial gradient: the maximum internal temperature reaches 64.027 °C, with the CPU region forming a high-temperature core area, which decreases radially toward the edges.

Based on the steady-state chassis temperature field, the ambient temperature data were applied as the initial thermal load for the transient thermal analysis of the motherboard. The transient temperature field at the motherboard mounting surface was extracted and used as the input boundary condition. The motherboard thermal simulation was performed using the thermal cycling profile shown in Figure 4a.

**Figure 8:**
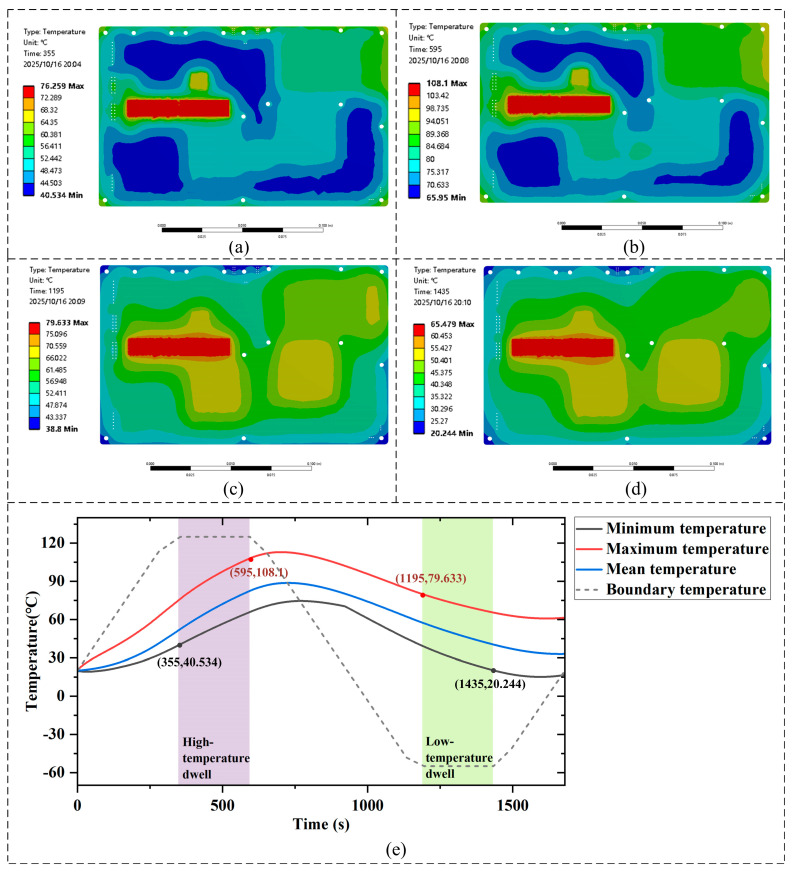
Motherboard temperature distribution: (**a**) minimum temperature during the high-temperature dwell. (**b**) maximum temperature during the high-temperature dwell. (**c**) maximum temperature during the low-temperature dwell. (**d**) minimum temperature during the low-temperature dwell. (**e**) motherboard temperature variation curve over time.

During the 125 °C high-temperature dwell, the motherboard reached a minimum temperature of 40.534 °C and a maximum of 108.1 °C, as shown in Figure 8a,b, respectively. During the −55 °C low-temperature dwell, the motherboard exhibited a maximum temperature of 79.633 °C and a minimum of 20.244 °C (Figure 8c,d). The relatively high maximum temperature during the low-temperature dwell occurs near the PCIe connector, where an additional heat flow of 1.2 W was applied to account for the local thermal loading. The overall temperature evolution of the motherboard over time is presented in Figure 8e, which clarifies the transient response during the entire thermal cycling process.

**Figure 9:**
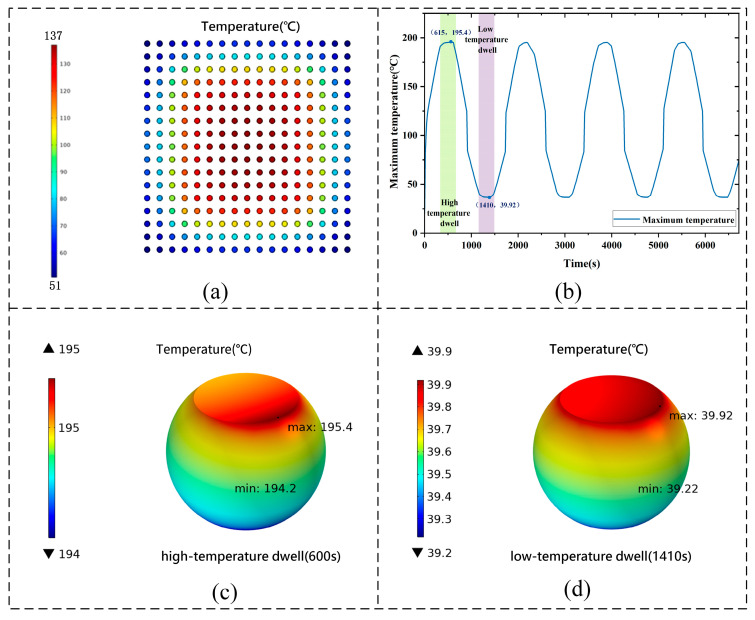
Temperature distribution of the 3A5000 chip: (**a**) Temperature distribution of the 17 × 17 solder joint array. (**b**) Time-dependent peak solder joint temperature curve. (**c**) Temperature distribution of central solder joints during high-temperature dwell. (**d**) Temperature distribution of central solder joints during low-temperature dwell.

Using submodeling techniques to map the motherboard temperature field, the 3A5000 CPU solder joint array exhibits similar mesoscopic-scale temperature gradient patterns. Figure 9a shows the temperature distribution cloud of the internal 17 × 17 solder joint array, where the central solder joints reach higher temperatures while the peripheral ones remain cooler. This occurs because heat dissipates more efficiently at the edges, whereas the central joints accumulate heat. In SnAgCu solder thermal failure studies, when the temperature approaches the solidus region, viscoplastic coupling and creep effects become significantly intensified. The temperature evolution curve in Figure 9b reveals the thermal response of the solder joints during the first four cycles: an initial steep temperature rise due to Joule heat accumulation, reaching the maximum heating rate within 315 s, followed by thermal equilibrium and maintenance of peak temperature during the dwell phase. The peak solder joint temperature reaches 195.4 °C during the isothermal dwell period, as shown in Figure 9c. During cooling, the solder joint temperature decreases with the environment, reaching a minimum of 39.92 °C at low-temperature dwell (−55 °C), as shown in Figure 9d. Thermomechanical coupling indicates that the interaction between internal chip heat sources and environmental thermal loads causes the actual device temperature to deviate significantly from the ambient baseline, producing a characteristic thermal gradient distribution.

**Figure 10:**
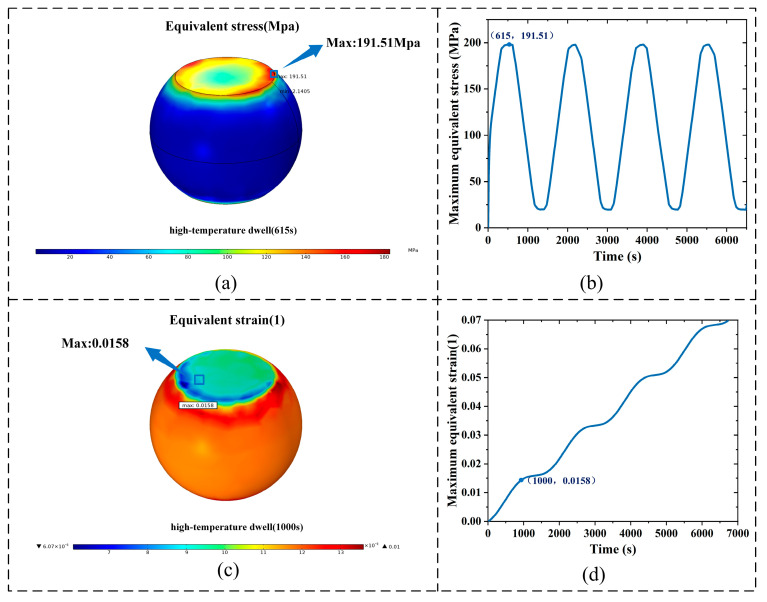
Solder joint stress–strain results: (**a**) Equivalent stress distribution at 615 s. (**b**) Time-dependent maximum solder joint stress curve. (**c**) Equivalent viscoplastic strain distribution at 1000 s. (**d**) Time-dependent maximum equivalent viscoplastic strain curve.

Figure 10a shows the equivalent stress distribution of the critical solder joint at the end of the first high-temperature dwell stage. The stress is mainly concentrated at the Cu/SnAgCu/Cu interface, with a maximum of 191.51 MPa, exceeding the yield strength and causing severe plastic deformation. Figure 10b illustrates the variation of maximum equivalent stress with time, consistent with the thermal cycling period: it increases during heating, peaks at the high dwell stage, and decreases during cooling, stabilizing at 20.36 MPa in the low dwell stage. Once the thermal stress surpasses the yield limit, plastic deformation accumulates, degrading mechanical performance. Figure 10c presents the equivalent viscoplastic strain distribution, with a maximum of 0.0158, consistent with the stress trend, identifying the joint as vulnerable. Figure 10d shows that viscoplastic strain increases stepwise with cycles, leading to cumulative damage and eventual fracture failure.

### 3.2. Dynamic Response Analysis

The thermal stresses generated during thermal cycling were applied as prestresses to perform a prestressed modal analysis of the 3A5000 motherboard. The objective was to determine its natural frequencies and corresponding mode shapes, thereby evaluating the dynamic response under actual operating conditions. Thermal expansion and temperature-dependent material properties alter the stiffness and boundary constraints, consequently affecting the distribution of natural frequencies. The first six natural frequencies at 125 °C were obtained as 1977.1 Hz, 3193.9 Hz, 3338.5 Hz, 4918.3 Hz, 6240.2 Hz, and 6932.2 Hz.

Random vibration response spectrum analysis was conducted to study the motherboard’s response under stochastic excitation, analyzing the statistical characteristics of physical quantities such as displacement, stress, and strain. Based on the modal results, a random vibration analysis was performed with fixed constraints applied at all through-hole locations to replicate operational boundary conditions.

**Figure 11:**
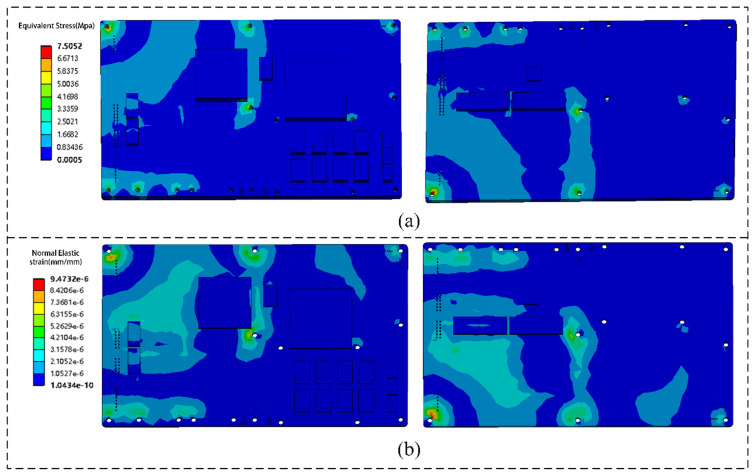
Stress and strain distribution of the 3A5000 motherboard: (**a**) Stress distribution on the top surface and bottom surface. (**b**) Strain distribution on the top surface and bottom surface.

Under random vibration, the stress distribution on the top and the bottom surfaces of the motherboard is shown in Figure 11a. Higher stresses occur at the through-hole surfaces constrained at the edges, with a maximum surface stress of 7.5052 MPa; the 3A5000 chip experiences higher stress than the 7A2000 chip. Strain distributions on the motherboard surfaces, shown in Figure 11b, indicate that stress and strain are primarily concentrated at the corners. Therefore, the four corners of the 3A5000 motherboard are susceptible to failure under vibration, suggesting that appropriate design measures should be taken when designing power modules to mitigate this risk.

Within the motherboard, solder interfaces and bonding wires are identified as structural weak points, with the failure modes of bonding wires closely related to the stress transfer characteristics of the solder joints. Given that the chip solder ball array forms a multilayer mechanical transmission path among the chip carrier, bonding wires, and substrate components, its interfacial coupling exhibits significant multi-physics interaction. Therefore, the focus here is on the chip solder ball array.

**Figure 12:**
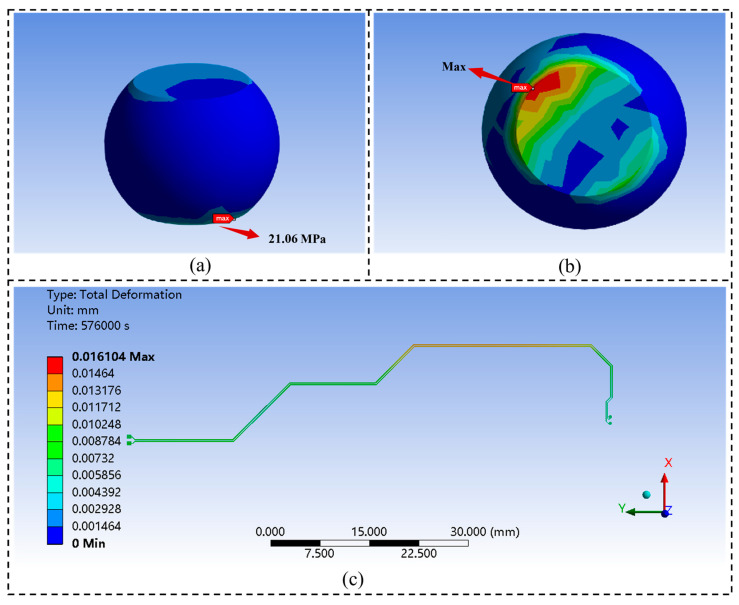
Solder joints and line deformation of the LS3A5000 chip: (**a**) Equivalent stress distribution of critical solder joints. (**b**) Normal strain distribution of critical solder joints. (**c**) Deformation of PCIe lines.

In the PSD response analysis of the solder ball array in the LS3A5000 chip, the stress distribution exhibits a distinct radial pattern. The solder balls near the geometric center experience relatively low stress levels, whereas the corner regions form stress concentration zones, with stress amplitude increasing nonlinearly along the radial direction. The maximum stress concentration occurs at the solder joints located near the PCB substrate interface at the array edges, as shown in Figure 12a, with a peak stress of 21.06 MPa. This region develops a typical three-dimensional coupled stress field due to the mismatch of the coefficients of thermal expansion (CTE) between the package and the substrate, combined with structural constraints. Figure 12b presents the normal strain distribution of the critical solder joint, where the maximum normal strain of the ball grid array (BGA) package structure reaches $2.0891\times 10^{-4}$. Furthermore, the deformed interconnect traces under the combined effects of thermal cycling and impact vibration were extracted. As shown in Figure 12c, the maximum deformation of the PCIe differential line reaches 16.104 μm, providing the deformed model for subsequent solder joint–interconnect electrical performance analysis.

### 3.3. Electrical Performance Analysis

#### 3.3.1. Electrical Failure Criterion

A mathematical model for solder joint resistance was developed, incorporating temperature-dependent material properties and void geometry. The temperature-dependent resistivity is given by the following:

(26) $$\rho (T)=\rho_{0}[1+\alpha (T-T_{0})]$$

where *ρ*(*T*) is the resistivity at temperature *T*, *α* is the temperature coefficient, and *T*_0_ is the reference temperature.

Under high-frequency conditions, current concentration due to the skin effect becomes significant. The skin depth *δ* is calculated as follows:

(27) $$\delta =\sqrt{\frac{\rho }{f\pi \mu }}$$

where *μ* is permeability, *f* is the signal frequency, and *ρ* is the resistivity.

Assuming a cylindrical solder joint, the resistance of a void-free solder joint is modeled as follows:

(28) $$R_{novoid}=\rho \frac{L}{A}=\frac{4\rho L}{\pi D^{2}}=\frac{4\rho_{0}[1+\alpha (T-T_{0})]L}{\pi D^{2}}$$

where *D* is the diameter and *L* is the height. Based on the geometric parameters, the initial resistance is calculated as *R*_0_ = 0.497 mΩ.

The failure criterion is defined as a 10% increase in resistance from its initial value [38]. Since resistance is inversely proportional to the conductive cross-sectional area:

(29) $$\frac{R_{novoid}}{R_{withvoid}}=\frac{S_{withvoid}}{S_{novoid}}$$

where *R_withvoid_* is the resistance with voids and *S_withvoid_* is the corresponding cross-sectional area. When the resistance increases by 10% (*R_fail_* = (1 + 10%) *R_novoid_*), the required cross-sectional area becomes the following:

(30) $$S_{withvoid}=\frac{10}{11}S_{novoid}$$

This corresponds to a 9.09% reduction in conductive area, equivalent to a void ratio of 9.09%. This microstructural degradation leads to significant impedance deterioration, with the measured resistance increasing by 10% from its original value, consistent with the inverse relationship between conductive area and resistance.

#### 3.3.2. Simulation of Void Evolution and Failure Time Prediction

**Figure 13:**
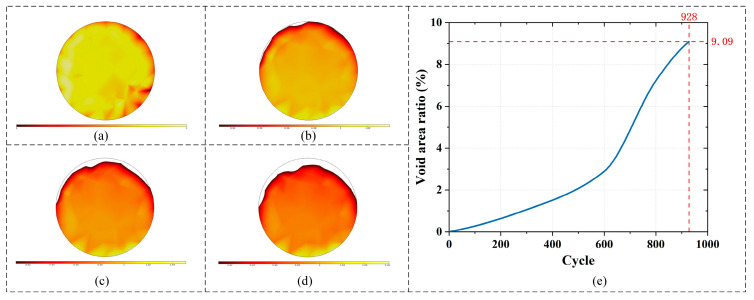
Cross-sectional views of void distribution in the solder joint: (**a**) initial state; (**b**) after 300 thermal cycles; (**c**) after 600 thermal cycles; (**d**) after 900 thermal cycles; and (**e**) the void area ratio against the number of thermal cycles.

The evolution of voids within the solder joint under thermal cycling was simulated to assess its impact on electrical reliability. Figure 13a–d presents the cross-sectional distribution of voids at the initial state and after 300, 600, and 900 cycles.

The results indicate a progressive expansion of voids along the circumference, leading to a continuous reduction in the conductive material. The void area ratio was quantified to be 1.06%, 2.89%, and 8.78% of the original joint area after 300, 600, and 900 cycles, respectively. A significant acceleration in void growth was observed between the 800th and 900th cycles, suggesting an expedited degradation phase as the damage accumulates.

The failure time was predicted based on the predefined electrical failure criterion. Figure 13e plots the void area ratio against the number of thermal cycles. The simulation data shows that the void ratio increases monotonically with the cycle count. The solder joint is predicted to reach the failure threshold—a void ratio of 9.09% corresponding to a 10% resistance increase—after 928 thermal cycles. This occurs at the high-temperature holding phase of that cycle, at a simulated time of *t* = 1,559,635 s, marking the onset of electrical failure.

#### 3.3.3. Electrical Performance

First, the PCB file of the motherboard was imported into Ansys SIwave to extract the three-dimensional wiring structure model, which was then imported into Ansys SpaceClaim for structural simplification. Subsequently, a multi-physics coupling analysis of the motherboard was performed in Ansys Workbench, where the transient temperature field of the motherboard mounting surface obtained from the chassis-level coupled analysis was applied as the boundary condition. Meanwhile, temperature cycling and mechanical shock loads were also imposed to capture the coupled thermal–mechanical response at the board level. Finally, the deformed PCIe traces and the corresponding solder joints were extracted, and an equivalent trace–solder joint model was constructed in CST for S-parameter and signal integrity analysis of high-speed interconnects.

**Figure 14:**
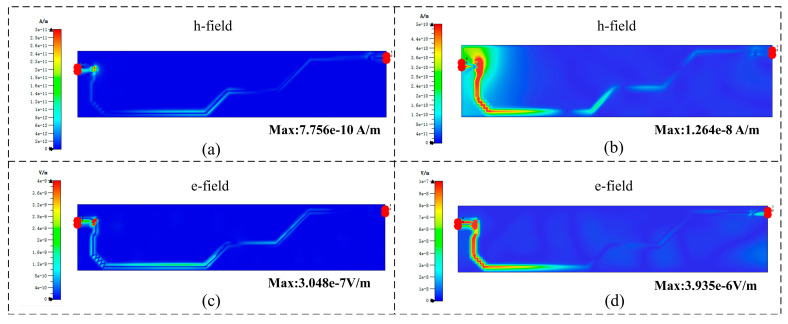
Electromagnetic coupling of PCIe_TX0_P/N: (**a**) Magnetic field distribution before the 928 cycles. (**b**) Magnetic field distribution after 928 cycles. (**c**) Electric field distribution before the 928 cycles. (**d**) Electric field distribution after the 928 cycles.

PCIe differential lines are highly sensitive to electromagnetic coupling, especially at high data rates, where even minor geometric variations can significantly impact signal integrity. Therefore, at the operating frequency, the electromagnetic coupling of the PCIe_TX0_P/N differential lines was analyzed before and after trace deformation, as shown in Figure 14. The results indicate that the coupling between the traces increases after deformation. The maximum magnetic field intensity rises from 7.756 × 10^−10^ A/m to 1.264 × 10^−8^ A/m (two orders of magnitude), while the maximum electric field intensity increases from 3.048 × 10^−7^ V/m to 3.935 × 10^−6^ V/m (one order of magnitude), leading to degraded signal transmission quality.

To further interpret the deformation–electrical coupling behavior, a quasi-static first-order sensitivity analysis of the coupling capacitance with respect to spacing *s* was introduced. Under the assumption of a parallel-field approximation, the coupling capacitance can be expressed as

(31) $$C=\frac{\varepsilon A}{s}$$

and its first-order derivative is given by

(32) $$\frac{\partial C}{\partial s}=-\frac{\varepsilon A}{s^{2}}$$

indicating that the capacitance decreases quadratically with increasing spacing. The relative variation can thus be approximated as

(33) $$\frac{\Delta C}{C}\approx -\frac{\Delta s}{s}$$

This relationship suggests that a local reduction in spacing intensifies the electric field and increases the coupling strength between differential lines. The observed field peaks correspond to the narrowest geometric regions of the traces, confirming that local deformation amplifies viscoplastic–electromagnetic interactions and directly affects signal integrity.

**Figure 15:**
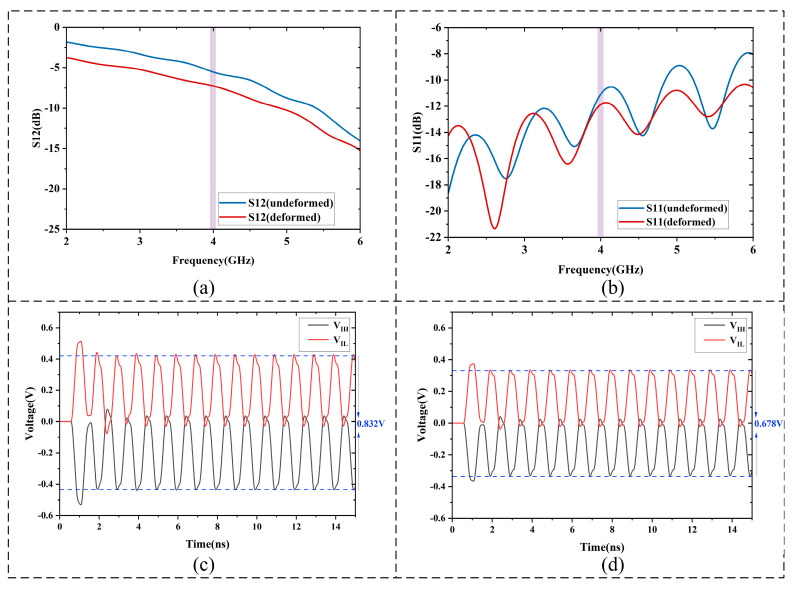
S-parameters and port voltage distributions of the line–solder joint equivalent model before and after deformation: (**a**) S12 before and after the 928 cycles. (**b**) S11 before and after the 928 cycles. (**c**) Port voltage distribution of PCIe_TX0_P/N before the 928 cycles. (**d**) Port voltage distribution of PCIe_TX0_P/N after the 928 cycles.

For the S-parameter analysis of the PCIe_TX0_P/N lines before and after deformation, as shown in Figure 15a,b, multiple electrical metrics were calculated to quantitatively evaluate the influence of geometric deformation on signal integrity. The insertion loss (IL) increased from 5.53 dB to 7.27 dB, showing a rise of 1.74 dB, indicating that signal attenuation across the entire frequency band became more pronounced but still remained within the PCIe specification limit of ≤8 dB. The return loss (RL) increased from 11.087 dB to 11.874 dB, with a variation of 0.788 dB, which also satisfies the consistency requirement of ≥10 dB. However, this phenomenon is primarily caused by the increased resistance of the solder joints, leading to partial energy absorption and dissipation as heat at the joint locations, rather than indicating an improvement in impedance matching of the high-speed signal traces. The mean differential imbalance increased from 0.032 to 0.064, implying that the symmetry of the differential signals was weakened, which may lead to stronger differential-to-common-mode noise coupling. The near-end crosstalk (NEXT) integral increased slightly from 0.003 to 0.004. Through this multi-metric analysis, the effects of geometric deformation on signal integrity are quantitatively revealed, providing substantial evidence for the degradation of the electrical performance of the PCIe_TX0_P/N differential pair.

Signal integrity analysis of the PCIe_TX0_P/N solder joint–transmission line equivalent model indicates that the standard peak-to-peak voltage swing of the differential pair is 800 mV, with the voltage difference between the positive and negative lines varying from −400 mV to 400 mV. The allowable safe voltage swing should be maintained within the range of 720 mV–800 mV, corresponding to a variation of less than 10% [38]. Simulation results show that before deformation, the peak-to-peak voltage at the receiver remains stable at 0.832 V, as shown in Figure 15c; after deformation, it decreases to 0.678 V, as shown in Figure 15d, representing a relative reduction of 18.51%. This amplitude degradation exceeds the ±10% tolerance range defined by the PCI Express^®^ Architecture PHY Test Specification, indicating non-compliance with the standard receiver voltage swing requirement and suggesting insufficient link margin for reliable transmission.

It should be noted that this amplitude degradation reflects an electrical performance deterioration at the physical layer, rather than an immediate functional communication failure. While the physical layer voltage swing falling outside the specification reduces the link margin and increases the likelihood of bit errors, the higher protocol layers (training, equalization, and retransmission mechanisms) may still temporarily maintain data transmission. Therefore, the observed degradation may result in insufficient link margin and degraded signal integrity, which can eventually lead to high-speed electrical failure after extended thermal cycling.

## 4. Design Guidelines

Based on the multiscale thermo–electro–mechanical analysis, several practical guidelines are proposed to enhance both structural reliability and signal integrity. Stress concentration mainly occurs at the BGA corners; local underfill or structural reinforcement can reduce plastic strain by about 15–25% and improve solder joint reliability. In addition, adopting graded materials or layout optimization between the array center and edges helps balance deformation and improves amplitude stability by approximately 5–8% during long-term thermal cycling. These measures provide practical design references for improving the durability and high-speed electrical performance of ruggedized computer systems.

## 5. Conclusions

This study addresses the multi-physics coupling problems of solder joints in microelectronic systems of ruggedized computers under complex service environments and proposes a multiscale modeling and analysis method. A model correlation study under ten thermal cycling conditions demonstrated an accuracy of 88.89%, confirming the validity and applicability of the proposed model. The approach enables multi-level coupled investigations ranging from the full computer level, board level, and solder joint level to the solder joint–interconnect microstructure level. The results indicate that, within the microelectronic systems of ruggedized computers, solder joints generate thermal stress and deformation due to temperature rises, while the thermal field alters electrical conductivity, inducing electromigration and concentration evolution. These, in turn, couple with stress gradients to form thermo–electro–mechanical multi-physics effects. The coupling among thermal, electrical, and mechanical fields significantly aggravates the risk of electrical failure in the microelectronic systems of ruggedized computers. The critical solder joints exhibit equivalent stress concentration at the metal interconnection interface, with a maximum value of 191.51 MPa, accompanied by pronounced plastic strain accumulation. Meanwhile, PCIe high-speed interconnect experienced a maximum deformation of 16.104 μm and a voltage amplitude reduction of approximately 18.51% after 928 thermal cycles, exceeding the normal operating range. These findings provide reliable theoretical support and engineering references for the optimized design and reliability assessment of ruggedized computers. The proposed model is primarily applicable to thermal cycling environments ranging from −55 °C to 150 °C, high-frequency vibrations of approximately 405 Hz, and mechanical shock conditions of up to 40 g. However, environmental factors such as acoustic noise, dust contamination, and humid–thermal conditions were not considered in the current modeling framework. Future work will further consider environmental factors such as acoustic noise, dust contamination and thermo–hygroscopic coupling, to enhance the model’s applicability under more complex service conditions.

## Figures and Tables

**Table 1:** Cross-Reference Table of Multi-Software Cross-Domain Data Mapping and Verification.

Analysis Layer	Input/Output Files & Parameters	Mapping/Verification Steps	Software
Geometric Layer	Parametric model files (.scdoc, .scscript); geometry simplification rules; reconstruction parameters	Geometry update; equivalence verification of solid–shell elements; coordinate system consistency check	Ansys SpaceClaim2023 R1
Thermal Analysis Layer	Temperature field results (.dat); boundary condition files; solver request files (.txt)	Mapping of temperature field onto structural mesh; thermal boundary consistency verification	Ansys Fluent2023 R1 Ansys Mechanical2023 R1
Thermo–Mechanical Analysis Layer	Nodal displacement and stress data (.mechdb); solver request files (.txt)	Mapping of deformation onto electromagnetic solver; solder joint stress–strain consistency verification	Ansys Mechanical2023 R1
Electromagnetic Analysis Layer	Electric and magnetic field distribution data (.txt); S-parameter data (.csv); solver request files (.txt)	Electromagnetic field–geometry consistency verification; S-parameter validation (S11, S21)	CST Studio Suite2023 AEDT2023 R1

**Table 2:** Material properties applied in the models.

Material	*ρ* (kg·m^−3^)	*Y* (GPa)	*ν*	CTE (K^−1^)	*K* (W·m^−1^·K^−1^)
Silicon	2329	131	0.3	2.8 × 10^−6^	150
Al_2_O_3_	2500	—	—	—	1.4
Copper	8960	127.7	0.31	1.7 × 10^−5^	393
Aluminum	2719	68.9	—	—	167
FR-4	1900	22	0.15	1.8 × 10^−5^	0.3
Sn–3.0Ag–0.5Cu (wt.%)	7390	as shown in Table 3	as shown in Table 3	as shown in Table 3	57.26
Vacuum	1.29	—	—	0.003	0.03

**Table 3:** Material properties of Sn–3.0Ag–0.5Cu (wt.%).

*T* (°C)	−40	−25	−5	25	50	75	100	125
*Y* (GPa)	46.89	45.79	44.38	43.25	41.43	39.45	36.85	34.59
*ν*	0.354	0.357	0.360	0.363	0.365	0.368	0.370	0.373
CTE (10^−5^·K^−1^)	2.36	2.40	2.43	2.50	2.61	2.67	2.73	2.79

**Table 4:** Anand parameters of Sn–3.0Ag–0.5Cu (wt.%) [19].

	*S*_0_ (MPa)	*Q/R* (K)	*A* (1·S^−1^)	*ξ*	*m*	*h_0_* (MPa)	*Ŝ* (MPa)	*n*	*a*
Sn–3.0Ag–0.5Cu (wt.%)	45.9	7460	5.87 × 10^6^	2	0.0942	9350	58.3	0.015	1.5

**Table 5:** Power Spectral Density (PSD).

Frequency/Hz	15	82.5	132.5	190	240.5	297.5	347.5	405
G acceleration (G·S^−2^)	0.01	0.3	0.3	0.15	0.15	0.075	0.075	0.0325

**Table 6:** Mechanical shock load.

Waveform	*P* g	*T*_D_ ms	Velocity Change Range m·s^−1^
Trailing Peak Sawtooth	40	11	*V_i_* ± 0.1*V_i_*

## Data Availability

The data presented in this study are available on request from the corresponding author. (Please specify the reason for restriction, e.g., the data are not publicly available due to privacy or ethical restrictions).

## Appendix A. Empirical Correlations for Thermal Boundary Conditions

### Appendix A.1. Natural Convection Heat Transfer

The natural convection heat transfer coefficient *h* is calculated using the empirical Rayleigh–Nusselt correlation, which relates the dimensionless Nusselt number Nu to the Rayleigh number Ra:

(A1) $$Nu=C\cdot Ra^{n}$$

where

(A2) $$Ra=\frac{g\cdot \beta \cdot (T_{s}-T_{amb})\cdot L^{3}}{\nu \cdot \alpha }$$

and g is the gravitational acceleration; *β* is the thermal expansion coefficient; *ν* is the kinematic viscosity; *α* is the thermal diffusivity; and L is the characteristic length of the surface.

For a vertical plate exposed to air under laminar free convection ($10^{4}<Ra<10^{9}$), the correlation proposed by Churchill and Chu [A2] is adopted:

(A3) $$Nu=0.68+\frac{0.670\cdot Ra^{1/4}}{{\left[1+{\left(0.492/Pr\right)}^{9/16}\right]}^{4/9}}$$

where Pr is the Prandtl number.

The convective heat transfer coefficient is then obtained as follows:

(A4) $$h=\frac{Nu\cdot k}{L}$$

where k is the thermal conductivity of the fluid.

For simplicity, in this study the relation is further expressed in the empirical form:

(A5) $$h=1.31\cdot {\left(T_{s}-T_{amb}\right)}^{0.33}$$

which is dimensionally consistent with Equation (A4) under the assumed conditions of air convection on a small vertical plate.

### Appendix A.2. Radiative Heat Transfer

The radiative heat flux is evaluated based on the Stefan–Boltzmann law [A3]:

(A6) $$q_{rad}=\varepsilon \cdot \sigma \cdot (T_{s}^{4}-T_{amb}^{4})$$

where *ε* is the surface emissivity (typically 0.8–0.95 for metal alloys), and $\sigma =5.670\times 10^{-8}\;W\cdot m^{-2}\cdot K^{-4}$ is the Stefan–Boltzmann constant.

### Appendix A.3. Applicability and Limitations

The above correlations are valid for natural convection in air near-atmospheric pressure.

The surface orientation is assumed to be vertical or nearly vertical.

The Rayleigh number range $10^{4}<Ra<10^{9}$ corresponds to laminar convection; for turbulent regimes, alternative coefficients should be adopted.

Radiation and convection are considered independent (non-participating medium assumption).
